# Multiple craniotomies in a single surgery — the resection of scattered brain metastases

**DOI:** 10.1007/s10143-023-01976-8

**Published:** 2023-03-15

**Authors:** Maximilian Bschorer, Franz L. Ricklefs, Thomas Sauvigny, Manfred Westphal, Lasse Dührsen

**Affiliations:** Department of Neurosurgery, University Medical Center Hamburg-Eppendorf, Hamburg, Germany

**Keywords:** Multiple craniotomies, Brain metastasis, Neurosurgery, Neuro-oncology

## Abstract

Patients with brain metastases (BM), who can benefit from resection of multiple scattered lesions, often will not be offered a procedure involving multiple craniotomies in one session due to the overall poor prognosis. However, carefully selected candidates may well benefit from the resection of multiple lesions using multiple craniotomies through a significantly shortened hospital stay, aggressive decompression, and rapid eligibility for adjuvant therapies. In this retrospective analysis, the records of patients, who were treated for multiple BM using one surgical session involving multiple craniotomies, were reviewed. A group of patients with multiple BM, whose surgery only involved one craniotomy, were assigned to a control group. Clinical and surgical characteristics, preoperative and postoperative Karnofsky Performance Scale (KPS), complication rate, preoperative tumor size, number of lesions, number of craniotomies, skin incisions, and intraoperative repositioning of patients were recorded. Thirty-three patients were included in the multiple-craniotomy group. Thirty patients underwent two craniotomies, while three cases involved three craniotomies. Seven patients (21%) were intraoperatively repositioned from a prone to a supine position, which required an average of 23.3 ± 9.3 min from wound closure to the following skin incision. Thirty-six patients with multiple BM and matching characteristics, who received only one craniotomy for the dominant lesion, served as the control group. No difference was detected in postoperative KPS (*p* = 0.269), complication rate (*p* = 0.612), rate of new postoperative neurological deficits (*p* = 0.278), length of intensive care unit (ICU) (*p* = 0.991), and hospital stay (*p* = 0.913). There was a significant difference in average preoperative tumor size (*p* = 0.002), duration of surgery (*p* < 0.001), and extent of resection (*p* = 0.002). In the age of personalized medicine, selected patient may benefit from a single surgery for BM using multiple craniotomies. This study shows no significant increase of the perioperative complication rate for surgeries with multiple craniotomies.

## Introduction

Recent advances in modern oncological therapy and the subsequent prolonged survival of cancer patients have led to a steady increase in the incidence of brain metastases (BM) [7]. Neurosurgical resection alone or in combination with modern chemotherapies, immunotherapies, stereotactic radiosurgery (SRS), and targeted therapies have improved the prognoses of patients diagnosed with BM. The role of neurosurgical interventions has also evolved in the direction of enabling other therapies by relieving symptoms [1]. Up to 80% of patients diagnosed with BM present with cerebral metastatic disease in a stage still suitable for local treatment modalities [13]. Characteristics such as comorbidities, tumor histology, and overall disease status play part in screening for surgical candidates [4]. In carefully selected cases, the resection of multiple BM causing neurological impairment by mass effect may rapidly improve the patients’ conditions when removed in a single surgical setting. Performing multiple craniotomies in such an approach may be warranted to shorten the time interval before further systemic treatment can be started.

Aggressive surgical therapy in patients with otherwise controlled extracranial disease can be beneficial to patients if surgical resection fits the overall oncological concept [2]. As outlined in the recommendations of the society of neuro-oncology (SNO), there are several factors that influence the selection of candidates for BM surgery. These factors include the reduction of tumor mass effect, absent histological diagnosis, and allowing for molecular analysis and consequently targeted molecular therapy [16]. Tumors located in the posterior fossa may be prognosis-limiting due to displacement of the fourth ventricle and subsequent hydrocephalus. The mass effect as well as the tumor-associated edema often requires high-dose glucocorticoid steroids. Surgery can provide rapid resolution of edema, relieve the mass effect, and enable steroid weaning [11]. Eloquent tumor localization and subsequent neurological deficits should also be taken into consideration. Surgery can allow patients to become eligible for adjuvant treatments, which has been associated with a better survival [14].

Contrary to the nihilistic approach taken to the situation of multiple BMs, one aspect of personalized medicine is offering selected patients with multiple BM and controlled or treatment-naïve extracranial disease the option of neurosurgical resection [5]. Even the need to resect scattered metastases through multiple craniotomies should warrant consideration. In that context, we present our experience with multiple craniotomies in single session as a safe, effective, and appropriate procedure with immediate consequences for further therapies.

## Materials and methods

Through a retrospective search of clinical records, we identified all patients, who underwent surgery using multiple craniotomies between 2016 and 2021 (intervention group, group A). Patients, who had multiple cerebral metastases and only underwent resection using only a single craniotomy either for a single large or symptomatic lesion or multiple lesions between 2020 and 2021, served as a comparator (control group, group B). Clinical records and surgical notes about the initial hospital stay and follow-up were reviewed. All surgeries were performed at the Department of Neurosurgery at the University Medical Center Hamburg-Eppendorf. All treatment decisions were supported by dedicated tumor boards for the respective entities unless emergency treatment was indicated.

All lesions, which were surgically removed in operations involving either a single or multiple craniotomies, met the following criteria: size (generally > 3 cm in diameter), eloquent location, extensive peritumoral brain edema, and the displacement of the cerebral fluid drainage apparatus causing hydrocephalus. Additional factors, which played a role in the decision-making process, were interdisciplinary tumor board decisions, the necessity to confirm histological diagnosis, or determine molecular tumor markers. While all patients in group B had multiple BM, a single craniotomy was sufficient to address the lesions that met criteria for surgical resection. The indication for multiple craniotomies in group A resulted from the necessity to simultaneously address multiple lesions for being symptomatic either directly by local compression or indirectly by causing any kind of obstructive hydrocephalus.

Inpatient and outpatient medical records and neuroimaging data were reviewed to assess clinical characteristics, performance status, tumor stage, number of sites of metastatic manifestation, and the degree of cerebral metastatic disease. Preoperative tumor size was determined by measuring the maximal diameter of the lesions in preoperative contrast-enhanced T1-weighed MRI sequence. The extent of resection was defined as either resection of all lesions spotted in the preoperative MRI, complete resection of the targeted lesions with remaining BM, and macroscopically incomplete resection of targeted lesions.

We conducted a PubMed literature search (search term: “multiple craniotomies,” date 04/2022) to identify publications on surgeries involving multiple craniotomies for BM in a single surgery. All statistics were done using the software R Studio version 1.4.1103. Statistical analysis was performed using Pearson’s chi-squared test, Fischer’s exact test, and the Mann–Whitney *U* test were used when appropriate. A *p* value of < 0.05 was indicative of a significant difference. The ethics committee of the physicians’ association of Hamburg approved this study. The registration number is 2021–300,167-WF. Data analysis was performed on anonymized data sets. The study was conducted in accordance with the ethical guidelines of the Helsinki Declaration.

## Results

Thirty-three patients (*n* = 33, male = 12, female = 21) with an average age of 59 years underwent surgery using multiple craniotomies in one session and were collected in group A. Thirty-six patients (*n* = 36, male = 11, female = 25, average age = 58 years) required only a single craniotomy in the presence of multiple lesions and were assigned to group B. The average number of intracranial lesions at the time of surgery was 4.7 ± 3.0 for group A and 4.6 ± 3.2 in group B (*p* = 0.995). In both cohorts, there is wide range of histological diagnoses. There is no difference in comorbidities and state of their metastatic disease, indicating relatively homogonous groups (Table 1).

**Table 1:** Patient population data: sites of extracranial tumor manifestation are either pulmonary, hepatic, lymphatic, renal, skeletal, cutaneous, adrenal glands, or the GI tract

Parameter	Multiple craniotomies (*n* = 33)	Single craniotomy (*n* = 36)	*p* value
Age			0.714
50 & older	22 (67%)	26 (72%)	
Under 50	11 (33%)	10 (28%)	
Sex			0.798
Female	21 (64%)	25 (69%)	
Male	12 (36%)	11 (31%)	
Anticoagulation drug therapy			0.563
Yes	6 (18.2%)	5 (13.9%)	
No	27 (81.8%)	31 (86.1%)	
Preoperative seizures			0.501
Yes	10 (30.3%)	7 (19.4%)	
No	23 (69.7%)	29 (80.6%)	
Diabetes			0.929
Yes	2 (6.1%)	2 (5.6%)	
No	31 (93.9%)	34 (94.4%)	
Preoperative neurological deficit			0.200
Yes	17 (51.5%)	12 (33.3%)	
No	16 (48.5%)	24 (66.6%)	
BMI			0.277
25 and under	14 (42%)	16 (44%)	
Over 25	19 (58%)	20 (56%)	
Origin of Primary tumor			0.995
Lung	12 (36%)	14 (39%)	
Skin (melanoma)	4 (12%)	3 (8%)	
Gastrointestinal tract	4 (12%)	5 (14%)	
Breast	6 (18%)	7 (19%)	
Other	7 (22%)	7 (19%)	
Interdisciplinary tumor conference			0.929
Yes	31 (94%)	2 (6%)	
No	34 (94%)	2 (6%)	
Sites of extracranial tumor manifestation			0.711
1	9 (27%)	14 (39%)	
2	12 (36%)	12 (33%)	
3	8 (24%)	6 (17%)	
4	3 (9%)	2 (6%)	
5	1 (3%)	2 (6%)	
Number of intracranial metastases			0.166
2	11 (33%)	10 (28%)	
3	6 (18%)	7 (19%)	
3–9	12 (36%)	17 (47%)	
10 +	4 (12%)	2 (6%)	
Preoperative tumor status			
Preoperatively confirmed histological diagnosis	25 (76%)	27 (75%)	0.942
Previous neurosurgical procedure	6 (18%)	4 (11%)	0.502
Previous cerebral radiation therapy	6 (18%)	5 (14%)	0.627

A score greater than one refers to the state in which the disease has also diagnosed at a site other than the primary tumor site and the brain. Statistical analysis performed using the Mann–Whitney *U* test, the chi-squared, or the Fischer’s exact test when appropriate

(*significant, *p* value < 0.05)

**Fig. 1:**
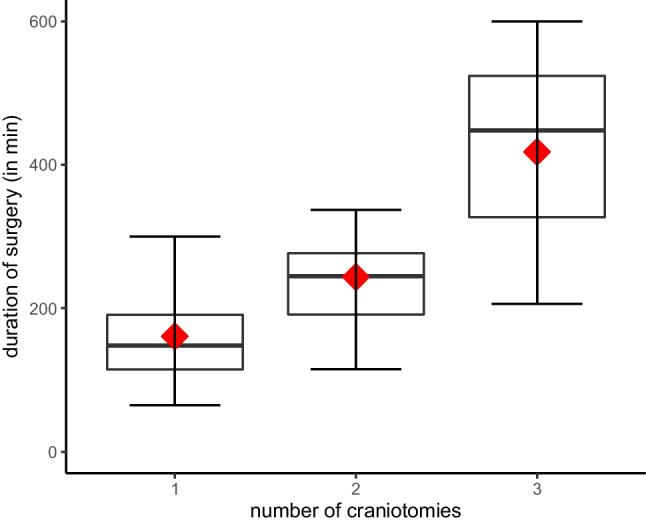
The duration of surgery shown by the number of craniotomies performed (*p* < 0.001). The mean is shown as a red diamond

**Fig. 2:**
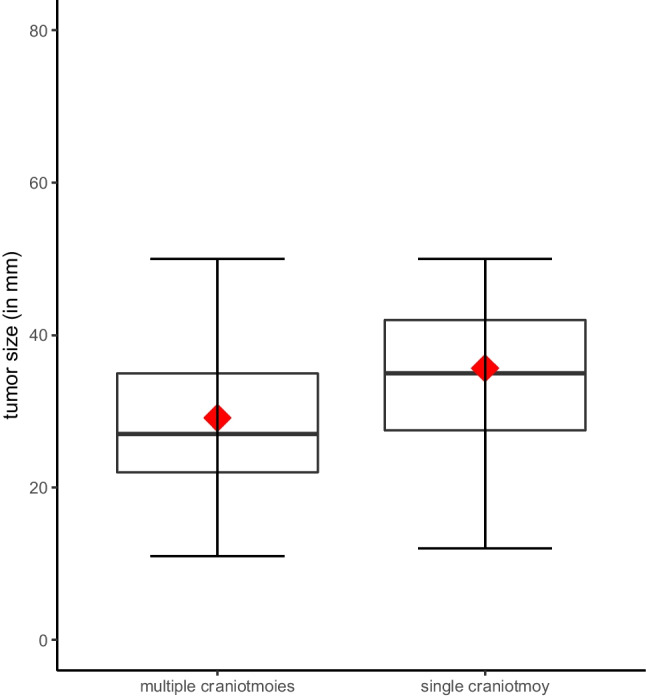
The preoperative tumor size (maximal diameter in mm) shown by group (*p* < 0.001). The mean is shown as a red diamond

Except for the three patients, who underwent three craniotomies, all other patients in group A underwent two craniotomies in single surgeries. Seven of the patients in group A had to be repositioned from a supine to a prone position during surgery. The duration of surgery in group A averaged 259.5 ± 95.8 min. In contrast, the average duration of surgery for group B was 160.8 ± 59.6 min, which indicates a significant difference (*p* < 0.001) (Fig. 1). The average time for intraoperative repositioning patients from prone to supine was 14.9 ± 11.5 min. The average size of the resected metastasis for the single craniotomy group was 35.7 mm, while the average metastatic lesion resected during surgery with multiple craniotomies was 29.1 mm (*p* = 0.002) (Fig. 2) (Table 2).

**Table 2:** Surgical data and statistical analysis performed using the chi-squared test or Fisher’s exact test when appropriate (*significant, *p* value < 0.05)

Characteristic	Multiple craniotomies (*n* = 33)	Single craniotomy (*n* = 36)	*p* value
Operative data			
Intraoperative ultrasound	19 (56%)	27 (75%)	0.201
Intraoperative repositioning of patient	7 (21%)		1
Intraoperative neuromonitoring	7 (21%)	2 (6%)	0.116
Number of resected metastases			< 0.001*
1		30 (83%)	
2	26 (79%)	6 (17%)	
3	6 (18%)	0 (0%)	
4	1 (3%)	0 (0%)	
Number of craniotomies			< 0.001*
1		36 (100%)	
2	30 (91%)		
3	3 (9%)		
Number of skin incisions			< 0.001*
1	2 (6%)	36 (100%)	
2	28 (85%)	0 (0%)	
3	3 (9%)	0 (0%)	
Location of resected metastases	*N* = 76	*N* = 49	
Frontal	17 + 3 × 2 (30%)	19 (25%)	0.011*
Parietal	12 + 2 × 2 (21%)	8 (20%)	0.197
Temporal	12 (16%)	1 × 2 (5%)	< 0.001*
Occipital	13 (17%)	5 (13%)	0.174
Infratentorial	10 + 1 × 2 (16%)	11 + 2 × 2 (38%)	0.872
Side of resection			0.034*
Left	7 (21%)	12 (33%)	
Right	11 (33%)	18 (50%)	
Both	15 (45%)	6 (17%)	

**Fig. 3:**
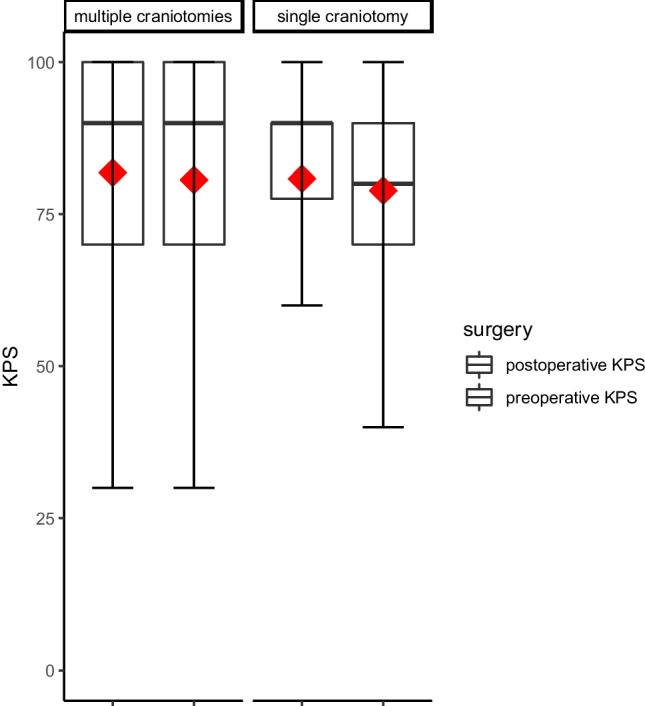
Comparison of preoperative Karnofsky Performance Scale (KPS) and postoperative KPS between the intervention group and the control group (*p* = 0.407). The mean is shown as a red diamond

In 11 cases of group A, the approach resulted in removal of all preoperatively diagnosed intracranial lesions, whereas that was neither the goal nor accomplished for the single craniotomies in group B, except for one case of juxtaposed lesions. There was no difference in the surgeries’ effect on the KPS, which improved by 1.2 from 80.6 to an average of 81.8 at the time of discharge for group A and from 78.9 to 80.8 for patients in group B (*p* = 0.407) (Fig. 3).

The average time until discharge was 14 ± 13.2 days for group A and 12.2 ± 6.9 days for group B (*p* = 0.913). Twenty-nine out of the 33 (88%) patients spent a single night at an intensive care unit in group A, while group B had a rate of 89% (*n* = 32) (*p* = 0.240). In both groups, there were two patients, who required emergency evacuation of postoperative hemorrhage (*p* = 0.929). Nine out of the 33 patients (27%) in group A developed new neurological deficits in the immediate postoperative phase with all of them showing improvement at the time of discharge. In group B, only 5 patients presented with new postoperative neurological deficits (14%). No statistical correlation could be observed (*p* = 0.233). There was no postoperative seizure or in-hospital mortality (Table 3). There was no difference in the rate of adjuvant treatment participation (chemotherapy, radiation therapy) (*p* > 0.05). The average days of follow-up were 300 ± 352 days for group A and 221 ± 183 days for group B. There was no difference in rate of surgical site infection between the two groups (*p* = 0.603). The results of our literature review on patient cohorts, who underwent multiple craniotomies in a single surgery, can be found in Table 4.

**Table 3:** Postoperative outcome: statistical analysis performed using the Mann–Whitney *U* test or the chi-squared test when appropriate (*significant, *p* value < 0.05)

Parameter	Group A (*n* = 33)	Group B (*n* = 36)	*p* value
Outcome			0.002*
Resection of all metastases	11 (33%)	1 (3%)	
Incomplete resection of metastases	0 (0%)	0 (0%)	
Remaining unresected metastases	22 (67%)	35 (97%)	
Postoperative Karnofsky Performance Index			0.407
Improvement	9 (27%)	15 (42%)	
Decline	7 (21%)	15(42%)	
No change	17 (52%)	6 (17%)	
Duration of ICU stay			0.991
1 day	29 (88%)	32 (89%)	
> 1 days	4 (12%)	4 (11%)	
Duration of hospital stay			0.913
Fewer than 10 days	15 (45%)	19 (58%)	
10 or more days	16 (55%)	17 (42%)	
Surgical complications			
Reoperation before discharge	5 (15%)	3 (8%)	0.466
Infection of surgical site within 6 months	2 (6%)	1 (3%)	0.603
Postoperative hemorrhage evacuation	2 (6%)	2 (6%)	0.929
Postoperative thromboembolic event	1 (3%)	5 (14%)	0.201
New neurological deficit	9 (27%)	5 (14%)	0.233
Postoperative seizure	0 (0%)	0 (0%)	1
In-hospital mortality	0 (0%)	0 (0%)	1
Postoperative treatment regimen			
Radiation therapy	27 (82%)	26 (72%)	0.410
Chemotherapy	24 (73%)	23 (64%)	0.728
Neurosurgical procedure after discharge	7 (21%)	4 (11%)	0.252
Mortality at follow-up			0.298
Alive	20 (61%)	28 (78%)	
Dead	13 (39%)	8(22%)	

*ICU* intensive care unit

**Table 4:** Literature overview of relevant PubMed publications on resection of multiple BM using multiple craniotomies in a single surgical setting (search term: “multiple craniotomies”)

Author, publication year	Study title	Study design	*n*	Result
Pollock et al. (2003) [10]	Properly selected patients with multiple brain metastases may benefit from aggressive treatment of their intracranial disease	Retrospective cohort study	6	This study demonstrates surgical feasibility of multiple craniotomies in a single surgery and showed an improved survival rate when comparing surgery and SRS with whole-brain radiation alone
Bindal et al. (1993) [2]	Surgical treatment of multiple brain metastases	Retrospective cohort study	21	A group of patients, who received resection of all metastases through multiple craniotomies, had a similar survival rate than a group of patients, who underwent complete resection of a single BM in cases of only one BM
Baker et al. (2017) [1]	Simultaneous multiple craniotomies in the management of multifocal malignant brain lesions: case reports	Case series	2	All patients showed an improvement of postoperative neurological outcome
Tanel et al. (2019) [15]	Simultaneous resection of multiple metastatic brain tumors with multiple keyhole craniotomies	Retrospective cohort study	20	The KPS improved in 71% of patients. The mean survival of these patients was 10.8 months
Paek et al. (2005) [9]	Reevaluation of surgery for the treatment of brain metastases: review of 208 patients with single or multiple brain metastases treated at one institution with modern neurosurgical techniques	Retrospective cohort study	17	Surgical resection improved neurological symptoms without an increased risk for complications

## Discussion

Patients with multiple BM present an oncological dilemma. Surgical options are frequently limited and patients reaching that disease stage are frequently branded as “terminal.” The approach, however, has to be much more differential, especially in times of personalized medicine. Patients with a dominant symptomatic lesion of a yet undiscovered primary may still have many “lines of therapy” ahead of them. Likewise, patients, who have not exhausted all options in a lengthy disease course with good quality of life, may well benefit from combined surgical/radiosurgical approaches [8]. In that context, we were concerned with patients, in whom a significant change of their disease course could be achieved when endeavoring on resection of multiple lesions in one session, even if it involved two or even three separate craniotomies. Screening for such surgical candidates for resection of multiple scattered BM is multifaceted and individualized in interdisciplinary discourse with radiotherapists and oncologists in dedicated tumor boards.

Submitting patients to aggressive surgical strategies must be weighed in perspective with the potential gain for the patient. The benefit for such surgical candidate is individual. Patients with two large and symptomatic but spread-out lesions will gain time and thus access to further therapies, even enabling them to benefit from experimental therapies or clinical trials based on KPS [17]. Others may gain time to have smaller or inaccessible lesions treated by radiosurgery or standard radiation therapy [11].

No matter the reason, the indication for such a surgical procedure was made in these select cases of multiple BMs, our study indicates that there is no increase in postoperative complication rate associated with an increased duration of surgery or multiple craniotomies. Emergency evacuation of postoperative hemorrhage as well postoperative infections occurred in similar rates. This study found no difference in KPS improvement in the comparison of the two groups, which is in agreement with the results of a 2005 study by Peak et al. [9]. The resected lesions of patients undergoing multiple craniotomies were mostly supratentorial metastases and smaller in diameter when compared to the lesions in group B, but a combination of supra- and infratentorial lesions were also safely approached. For these cases, the infratentorial lesions was approached first to avoid putting patients into the prone position after resection of supratentorial metastases. Also, optimal positioning for each lesion is mandatory, even if two supratentorial lesions, refixation, and redraping are necessary to make small navigated linear incisions.

Our series expands on earlier studies by Bindal, which found no difference in surgical complication rates of surgeries involving multiple craniotomies regardless of number of craniotomies performed. The authors also found no difference in survival between patients, who underwent total resection of multiple BM using multiple craniotomies and patients, who received total resection of a single BM [2]. The control of systemic disease generally determines survival. In that context, however, it appears of major importance to stress the safety of aggressive surgical treatment of patients presenting with BM using multiple craniotomies as systemic disease control is drastically improving for most tumor entities. Thus, patients develop BMs later in their disease course, and the number of patients with BMs is steadily increasing [6]. Aggressive surgical treatment in these patients has shown to increase survival if patients can subsequently be in enrolled in adjuvant treatment protocols [10]. Baker et al. published a series of 20 patients undergoing such procedures, and the authors showed promising postoperative results with an increase of the average postoperative KPS [1]. In a different context, multiple craniotomies in a single surgery for multifocal glioblastoma have also been associated with a comparable outcome and rate of complication as for resection using a single craniotomy supporting the concept of technical safety [3]. We strongly advocate the concept that in selected cases the surgical strategy of resecting scattered BM using an approach involving multiple craniotomies can be beneficial and safe.

Comparing this study’s cohort with a cohort of patients with multiple BM warranting resection, which were resected in multiple surgical sessions, would be interesting. However, at our institution, performing multiple craniotomies in a single session has been implemented as the standard protocol for resecting multiple brain lesions. While previous studies have observed an increased risk for postoperative complications for neurosurgical procedures, which lasted for more than 4 h, we did not observe such an increase in postoperative complications despite the significantly longer surgery durations [12]. Prospective studies with larger cohorts need to investigate, whether this surgical fits into the overall oncological concept and increase overall all morbidity and mortality. This study shows that patients with multiple BM may benefit from resection of more than one lesion in a single surgery. While the indication for such a surgical strategy is highly individualized, this study indicates no increased perioperative risk in the carefully selected candidates, who met the criteria to undergo procedure. Modern management of systemic oncological disease and targeted therapy warrants for individualized neurosurgical interventions, and in selected patients, the resection of multiple BM using resection of multiple scattered lesions may be warranted.

The retrospective nature of this study and the size of the study population limit the power of this study. Only limited conclusions about the overall neuro-oncological benefit on morbidity and mortality can be drawn from this comparison of the two groups because of the highly individualized surgical indications of patients undergoing multiple craniotomies in a single surgery. The size of the craniotomies may be a confounding variable in this study, especially since the size of the BM in group B was significantly larger compared to group A. We can only report on the technical feasibility of this procedure; however, this study indicates that there is no overall increase in complication rate for similar patient cohorts if surgery involved more than one craniotomy compared to a single craniotomy.

## Data Availability

Not applicable.
